# Upright CPR: A novel approach to delivering chest compressions to a seated casualty in a diving bell

**DOI:** 10.1016/j.resplu.2024.100707

**Published:** 2024-07-17

**Authors:** Andrew Tabner, Graham Johnson, Nicholas Tilbury, Alistair Wesson, Gareth D. Hughes, Rebecca Elder, Mari Östin, Philip Bryson

**Affiliations:** University Hospitals of Derby and Burton NHS Foundation Trust, Royal Derby Hospital, Uttoxeter Road, Derby DE22 3NE, UK; University of Nottingham School of Medicine, Queen’s Medical Centre, Nottingham NG7 2UH, UK; University Hospitals of Derby and Burton NHS Foundation Trust, Royal Derby Hospital, Uttoxeter Road, Derby DE22 3NE, UK; University of Nottingham School of Medicine, Queen’s Medical Centre, Nottingham NG7 2UH, UK; University Hospitals of Derby and Burton NHS Foundation Trust, Royal Derby Hospital, Uttoxeter Road, Derby DE22 3NE, UK; Independent, UK; University Hospitals of Derby and Burton NHS Foundation Trust, Royal Derby Hospital, Uttoxeter Road, Derby DE22 3NE, UK; North West School of Anaesthesia, Manchester, UK; DEEP Research Labs Ltd., Unit 4, Portside Park, Kings Weston Lane, Avonmouth BS11 8AR, UK; TAC Healthcare, Wellheads Crescent, Aberdeen AB21 7GA, UK

*Dear editor,*

A diving bell is extremely cramped, and often lacks a flat surface on which CPR can be performed^1^; it may therefore be necessary to provide chest compressions to a seated casualty, with the only evidenced methods being either seated knee-to-chest or mechanical CPR.^2^, ^3^ The evidence to support “head-up CPR” is not yet sufficient to encourage its adoption when supine CPR is practical^4^; these are therefore methods intended for use only when it is impossible to lie a casualty supine.

Our concurrent article in this edition describes the development of an algorithm for the management of cardiorespiratory arrest in a diving bell^5^; the divers involved in algorithm development proposed an alternative approach for delivering chest compressions in this setting. Best described as “upright CPR” (but affectionately termed “The Dunoon Method” in honour of its development location), it involves the provider either standing or in a high-kneeling position (Fig. 1), rear foot braced against the diving bell wall, providing compressions using their hands placed on the casualty’s chest with fingers cephalad.

**Fig. 1:**
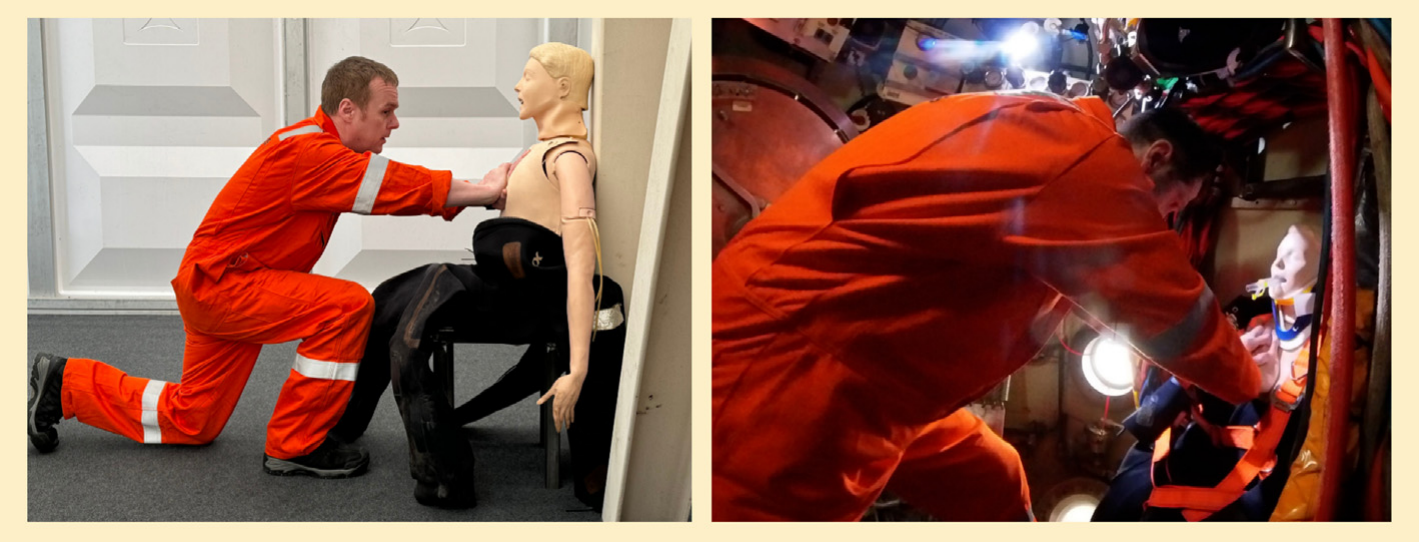
Upright (“Dunoon”) CPR simulated on land (left) and in a diving bell (right).

We undertook an exploratory evaluation of this technique to ascertain whether it could be considered for inclusion in the algorithm. Eleven divers each delivered 2 min of upright chest compressions to a seated Resusci Anne QCPR manikin. They reported that it was deliverable, but found the ergonomics difficult due to a low chair position (Fig. 1) and a lack of bracing for their rear foot. Nevertheless, manikin metrics were encouraging: median compression depth 57 mm (range 49–64); percentage of compressions to depth 53% (6–100); adequate recoil 89% (4–100); rate 116 bpm (72–131).

The technique was therefore further evaluated inside a diving bell, with 7 randomly allocated pairs of divers each delivering 4 min of CPR (including ventilations) and alternating provider at their discretion. Compression depth was 49 mm (41–52), with 53% of compressions to depth (26–99), adequate recoil in 47% (24–96), rate 114 bpm (108–125) and with an average pause duration of 3 s (3–4).

Eleven divers reported that upright CPR felt significantly more sustainable than seated knee-to-chest CPR in the bell; 2 felt that both methods were equally sustainable. The latter two were amongst the tallest in the group (∼198 cm), and it is likely that ergonomic factors contribute to the relative perceived sustainability of the two techniques.

The proposal of a novel chest compression technique was an unanticipated outcome of the week, and its evaluation should be considered exploratory. Divers were not “trained” in this technique; they were contributing to its development. Whilst compression metrics do not meet the standard targets for depth and recoil, the technique is being delivered in a non-standard environment; it may represent the best achievable manual technique in the circumstances.

Further work is required to refine the technique, evaluate the impact of ergonomic factors on its deliverability, and ascertain how it compares to standard CPR when delivered by trained providers of differing sizes. However, it currently represents an acceptable alternative to seated knee-to-chest CPR in a diving bell, and may have utility in other settings.

## Conflicts of interest

The overall project was funded through donation from a number of commercial organisations listed in the funding section of this manuscript.

Representatives from several organisations were present in their professional roles to offer their personal experiences, opinions and expertise during the refinement phase.

The Professional Diving Academy, Dunoon, and Submarine Manufacturing and Products Limited, Preston, created the simulation complex for the refinement phase at no cost to the research budget.

None of the above individuals or organisations have had any control or influence over project design, delivery, data collection, data analysis, data interpretation or publication.

All research members and industry representatives participated either voluntarily, or as part of their normal employment; no attendees received extra payments for participation.

## Funding statement

The project would not have been possible without generous support from the following organisations:

Professional Diving Academy, Submarine Manufacturing and Products, Boskalis, NUI, BP, DFS Diving, Equinor, IMCA, K-Subsea, KD Marine, Rever, RockSalt Subsea, Shelf Subsea, Subsea 7, TechnipFMC, Total Energies, Unique Hydra (PTY) Ltd, Well-Safe Solutions.

These funders had no role in the design or implementation of the protocol or the decision to publish.

International SOS and TAC Healthcare sponsored the work of one of the authors: Dr P Bryson.

## Approvals

This overall study was sponsored by the University Hospitals of Derby and Burton NHS Foundation Trust, reference number DHRD/2018/021.

This study was approved by the Health Research Authority, IRAS reference 247680.

No ethical approvals were required.

## CRediT authorship contribution statement

**Andrew Tabner:** Conceptualization, Data curation, Formal analysis, Funding acquisition, Investigation, Methodology, Project administration, Resources, Supervision, Validation, Visualization, Writing – original draft, Writing – review & editing. **Graham Johnson:** Conceptualization, Data curation, Formal analysis, Funding acquisition, Investigation, Methodology, Project administration, Resources, Supervision, Validation, Visualization, Writing – original draft, Writing – review & editing. **Nicholas Tilbury:** Investigation, Writing – review & editing, Methodology. **Alistair Wesson:** Investigation, Methodology, Writing – review & editing. **Gareth D. Hughes:** Data curation, Investigation, Writing – review & editing. **Rebecca Elder:** Investigation, Writing – review & editing. **Mari Östin:** Investigation, Writing – review & editing. **Philip Bryson:** Conceptualization, Funding acquisition, Investigation, Methodology, Project administration, Writing – review & editing.

## Declaration of competing interest

The authors declare that they have no known competing financial interests or personal relationships that could have appeared to influence the work reported in this paper.
